# The anti-cancer effects and mechanisms of *Scutellaria barbata* D. Don on CL1-5 lung cancer cells

**DOI:** 10.18632/oncotarget.22677

**Published:** 2017-11-27

**Authors:** Chin-Chuan Chen, Chun-Pin Kao, Mei-Miao Chiu, Shu-Huei Wang

**Affiliations:** ^1^ Graduate Institute of Natural Products, Chang Gung University, Taoyuan, Taiwan, Chinese Herbal Medicine Research Team, Healthy Aging Research Center, Chang Gung University, Taoyuan, Taiwan; ^2^ Tissue Bank, Chang Gung Memorial Hospital, Taoyuan, Taiwan; ^3^ Department of Nursing, Hsin Sheng Junior College of Medical Care and Management, Taoyuan, Taiwan; ^4^ Department of Medicine, Mackay Medical College, New Taipei, Taiwan, Republic of China; ^5^ Department of Anatomy and Cell Biology, College of Medicine, National Taiwan University, Taipei, Taiwan

**Keywords:** Scutellaria barbata D. Don, apoptosis, ER-stress, P38/MAPK, G2/M phase arrest

## Abstract

Lung cancer, with a poor prognosis and resistance to chemotherapy, is the most common malignant tumor and has the highest mortality rate worldwide. *Scutellaria barbata* D. Don (SB), which is derived from the dried whole plant of Labiatae, is a well-known anti-inflammatory and anti-cancer herb. The aim of this study was to examine the anti-cancer effects and precise regulatory mechanisms of SB in CL1-5 lung cancer cells. In an *in vitro* assay, we found that the anti-tumor mechanism of SB was due to P38/SIRT1-regulated cell apoptosis through G2/M phase arrest and ER stress-, intrinsic mitochondrial-, and extrinsic FAS/FASL-mediated pathways. Autophagy also plays a key role in SB-induced CL1-5 cell cytotoxicity. In addition, SB exerts additive effects with etoposide or cisplatin in lung cancer cells. In an *in vivo* assay, we found that SB *significantly* reduces tumor size with decreased proliferation and angiogenesis, as well as increased apoptosis and autophagy in CL1-5 tumor-bearing mice. These findings provided experimental evidence for the application of SB in the treatment of lung cancer.

## INTRODUCTION

Lung cancer is the one of most frequently diagnosed cancers and has the highest mortality rate worldwide [1]. Currently, radiotherapy and chemotherapy are still the standard therapy method, but only a modest increase in survival rate is found after the application of standard therapy in lung cancer patients due to resistance to radiotherapy and chemotherapy [2]. Thus, the development of effective and therapeutic agents is important and urgent for lung cancer treatment.

SIRT1 is a NAD+-dependent protein deacetylase, and its function is to regulate cell growth, apoptosis, aging and cellular metabolism through deacetylation and regulation of p53, PGC-1α, FOXO, NF-κB, histone and DNA damage response proteins [3-8]. SIRT1 is highly expressed in various types of cancer, including breast, colon, and non-small cell lung cancer. Activation of SIRT1 promotes tumor cell migration and lung metastasis of breast cancer in mice [9]. A previous study showed that SIRT1 expression is negatively associated with chemotherapy response and the prognosis of lung cancer [10-12]. SIRT1 induces cancer cell apoptosis via the modulation of p53 and FOXO [6]. Downregulation SIRT1 expression or inhibition of SIRT1 activation induces apoptosis and enhances radiation sensitization in lung cancer cells [12]. These studies indicate that the oncogenic characteristic of SIRT1 plays an important role in lung cancer progression. Therefore, SIRT1 inhibition might be an effective therapeutic strategy for the treatment of lung cancer.

**Figure 1:**
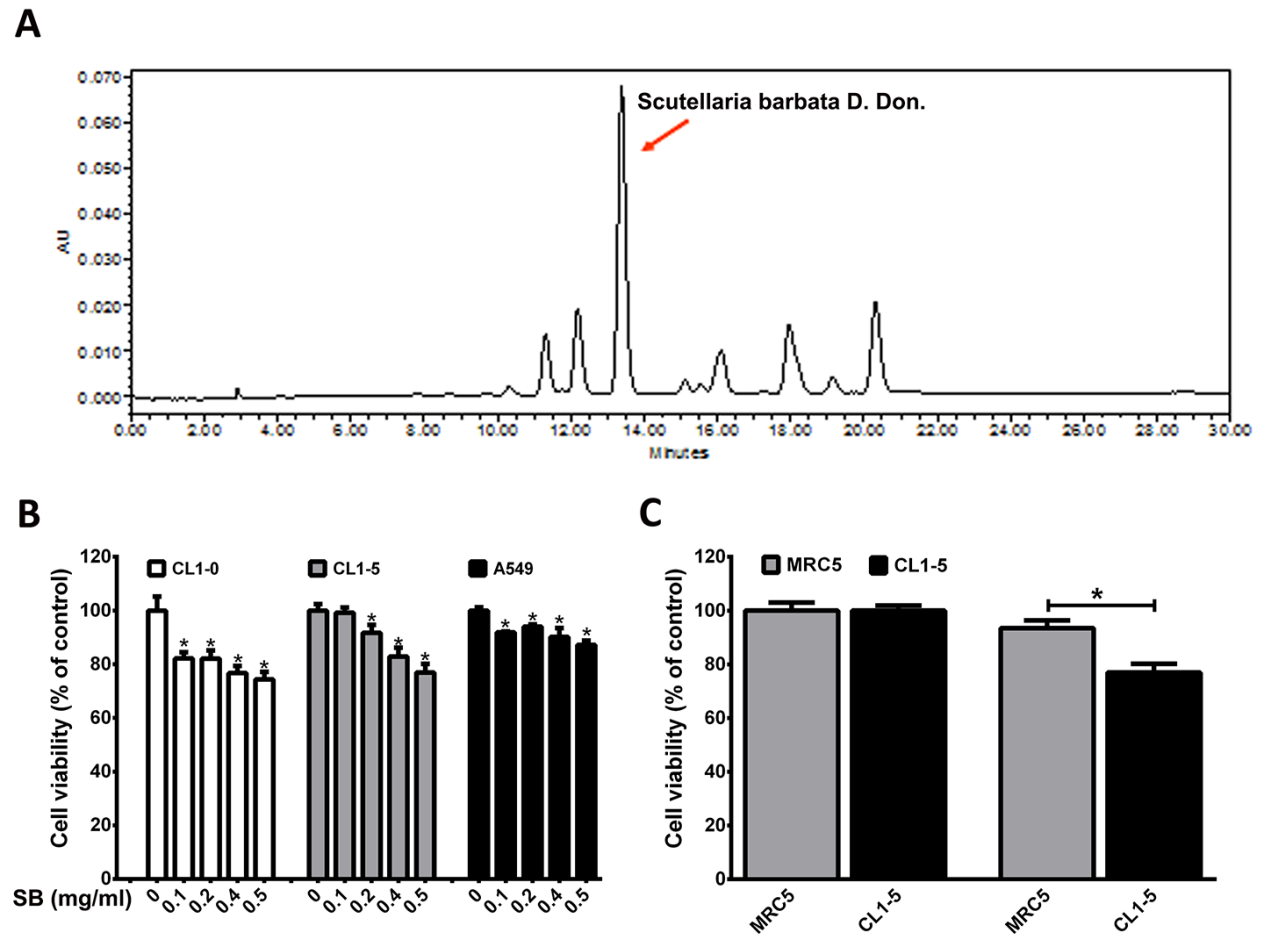
Cytotoxicity of various lung cancer cells and normal lung MRC5 cells was monitored by MTT assay **(A)** HPLC chromatogram of SB. **(B)** CL1-0, CL1-5, and A549 cells were treated with different concentrations of SB for 24 h. ^*^P<0.05 vs. the untreated group. **(C)** MRC5 and CL1-5 cells were treated with 0.5 mg/ml SB for 24 h. ^*^P<0.05. N=6-16. Data are represented as the mean±SEM.

*Scutellaria barbata* D. Don (SB) (Figure 1A), which is derived from the dried whole plant of Labiatae, is a well-known herb and drug in traditional Chinese Medicine where it is used as an anti-inflammatory herb. In recent years, some researchers have demonstrated that SB has significant antitumor activity in breast cancer [13, 14], colorectal cancer [15-18], hepatocarcinoma [19-21], uterine leiomyoma [22, 23], cervix cancer [24], skin cancer [25] and lung cancer [26-28]. However, the precise mechanism of the anti-tumor effect of SB in lung cancer is not yet clear. Therefore, the aim of the study was to investigate the anti-lung cancer molecular mechanisms of SB. In this study SB showed *in vitro* and *in vivo* anti-tumor activity through multiple pathways. SB induced lung cancer cell death through cell cycle arrest, apoptosis and autophagy. We further showed that the induction of G2/M phase arrest and apoptosis was mediated by the P38/SIRT1 signaling pathway. In addition, SB increased the therapeutic effects of etoposide and cisplatin treatment in lung cancer cells. These data indicated that SB may be a potential and effective anti-lung cancer drug.

## RESULTS

### SB shows selective cytotoxicity on cancer but not normal lung cells

To determine the growth inhibitory effect of SB on lung cancer cells, an MTT assay was performed. As shown in Figure 1B, SB caused a marked growth inhibitory effect on CL1-5, CL1-0, and A549 human lung cancer cell lines in a dose-dependent manner, and the growth inhibitory effect was not observed in normal lung fibroblasts (MRC5) (Figure 1C). These data suggested that SB causes lung cancer cell cytotoxicity.

### SB induces CL1-5 cell mitotic arrest

**Figure 2:**
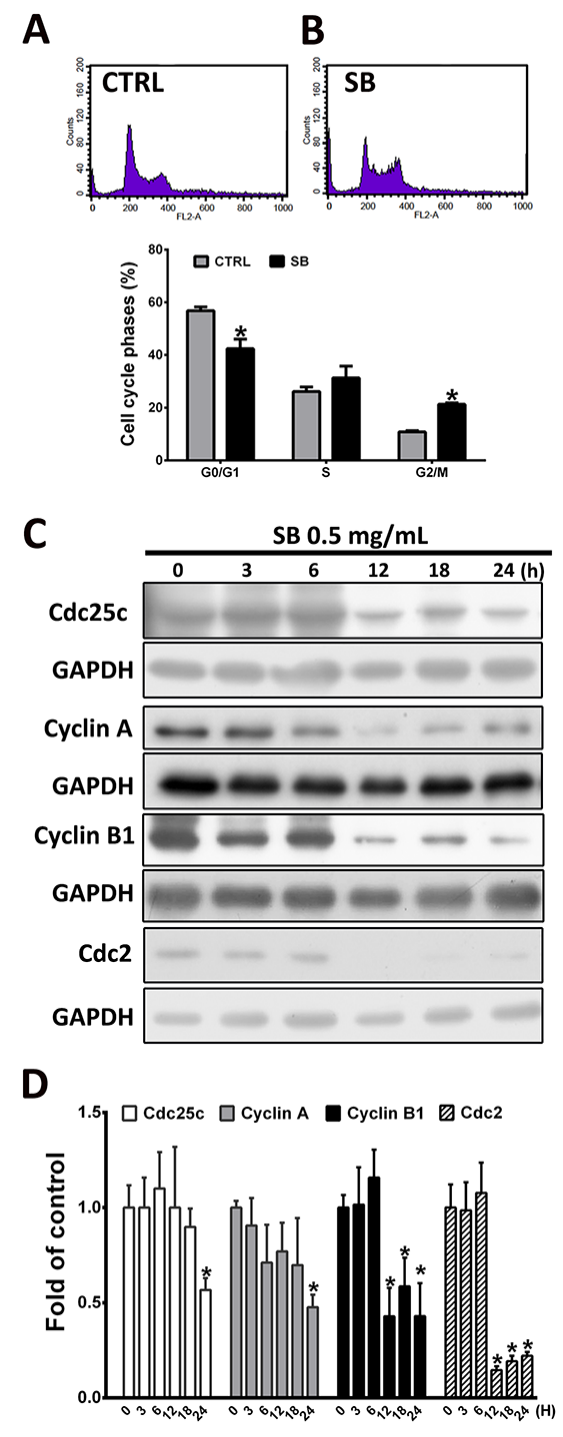
SB induces CL1-5 cell arrest in the G2/M phase **(A-B)** CL1-5 cells were treated with 0.5 mg/ml SB for 24 h, and the cell cycle distribution was determined by flow cytometry. **(C)** Western blot analysis of G2/M transition-related proteins after 0.5 mg/ml SB treatment of CL1-5 cells for different times. **(D)** Quantification of the western blot analysis. Data are represented as the mean±SEM. N=4-6. ^*^P<0.05 vs. the untreated group (CTRL).

To determine whether the effects of SB on CL1-5 cell growth and survival were due to cell cycle arrest, flow cytometry was performed. As shown in Figure 2A-B, SB treatment increased the proportion of CL1-5 cells in the G2/M phase from 10.82±0.45% to 21.3±0.55% compared with the vehicle control group (*P*<0.05), and this increase was accompanied by a decrease in cells in the G1 phase of the cell cycle. These data showed that SB promotes cell growth inhibition by inducing G2/M phase arrest in CL1-5 cells. The formation of complexes with regulatory cyclins plays an important role for cells to enter into mitosis [29]. To determine the mechanisms underlying CL1-5 cell mitotic arrest and proliferation inhibition by SB treatment, we examined the effect of SB on cell cycle regulatory proteins, including cyclins, by western blot analysis. As shown in Figure 2C-D, treatment with 5 mg/ml SB for various times (0, 3, 6, 12, 18 and 24 h) caused a significant reduction in the protein levels of Cdc25C, cyclin A, cyclin B1, and Cdc2 in a time-dependent manner. These data showed that SB inhibits mitotic entrance by reducing the levels of Cdc25C, cyclin A, cyclin B1, and Cdc2 in CL1-5 cells.

### SB-induced CL1-5 cell apoptosis

**Figure 3:**
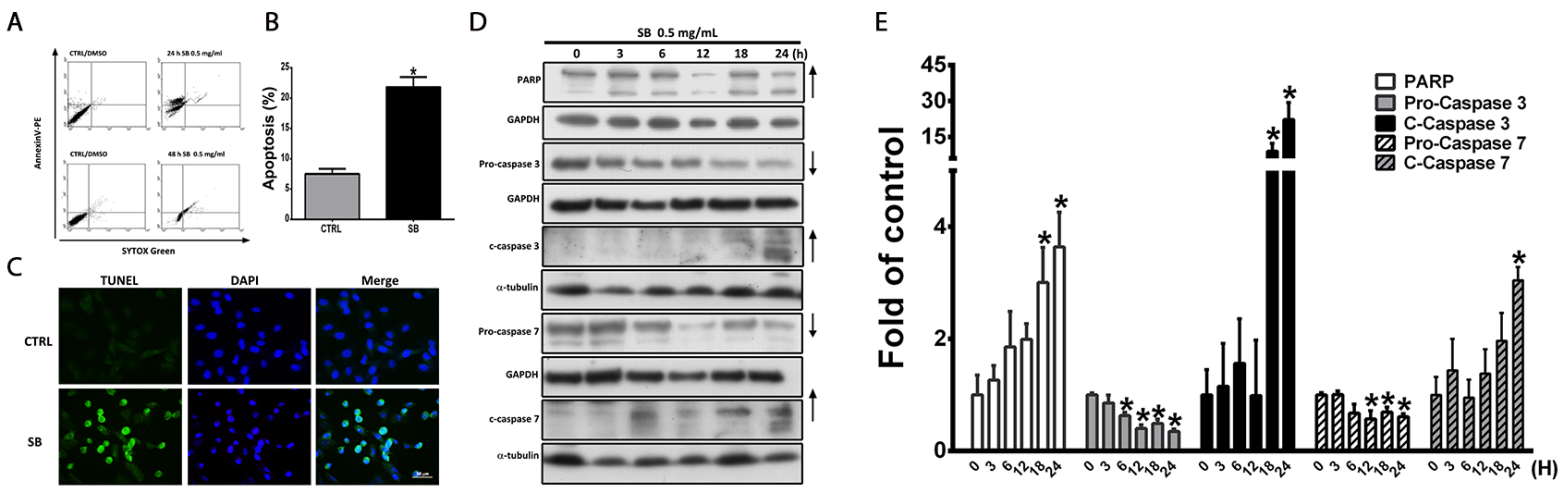
SB induces CL1-5 cell apoptosis **(A)** CL1-5 cells were treated with 0.5 mg/ml SB for 24 and 48 h, and the apoptotic cells were determined by flow cytometry. **(B)** Histogram shows the ratio of apoptotic cells induced by SB treatment for 24 h. **(C)** Representative immunofluorescence images of TUNEL-positive (green) CL1-5 cells at 24 h after exposure to 0.5 mg/ml SB. The cells were counterstained with DAPI (blue) to show all cell nuclei. Scale bar=50 μm. **(D)** Western blot analysis of apoptosis-related proteins after 0.5 mg/ml SB treatment of CL1-5 cells for different times. **(E)** Quantification of the western blot analysis. Data are represented as the mean±SEM. N=4-9. ^*^P<0.05 vs. the untreated group (CTRL).

To further elucidate the mechanisms of SB-induced CL1-5 cell death, CL1-5 cells were treated with 0.5 mg/ml SB for 24 h and then subjected to Annexin V/PI staining, TUNEL staining and western blotting. As shown in Figure 3A-B, Annexin V/PI staining showed a significant increase in apoptotic cells following treatment with SB for 24 h (*P*<0.05). Similar data were observed for TUNEL staining (Figure 3C). Western blot analysis was used to investigate the apoptotic proteins in SB-treated CL1-5 cells. As shown in Figure 3D-E, SB treatment induced the proteolytic cleavage of procaspase 7 and procaspase 3, as well as PARP expression. These data demonstrated that SB treatment effectively induces CL1-5 cell apoptosis.

### SB induces pro-apoptotic endoplasmic reticulum (*ER*) stress signaling pathway

**Figure 4:**
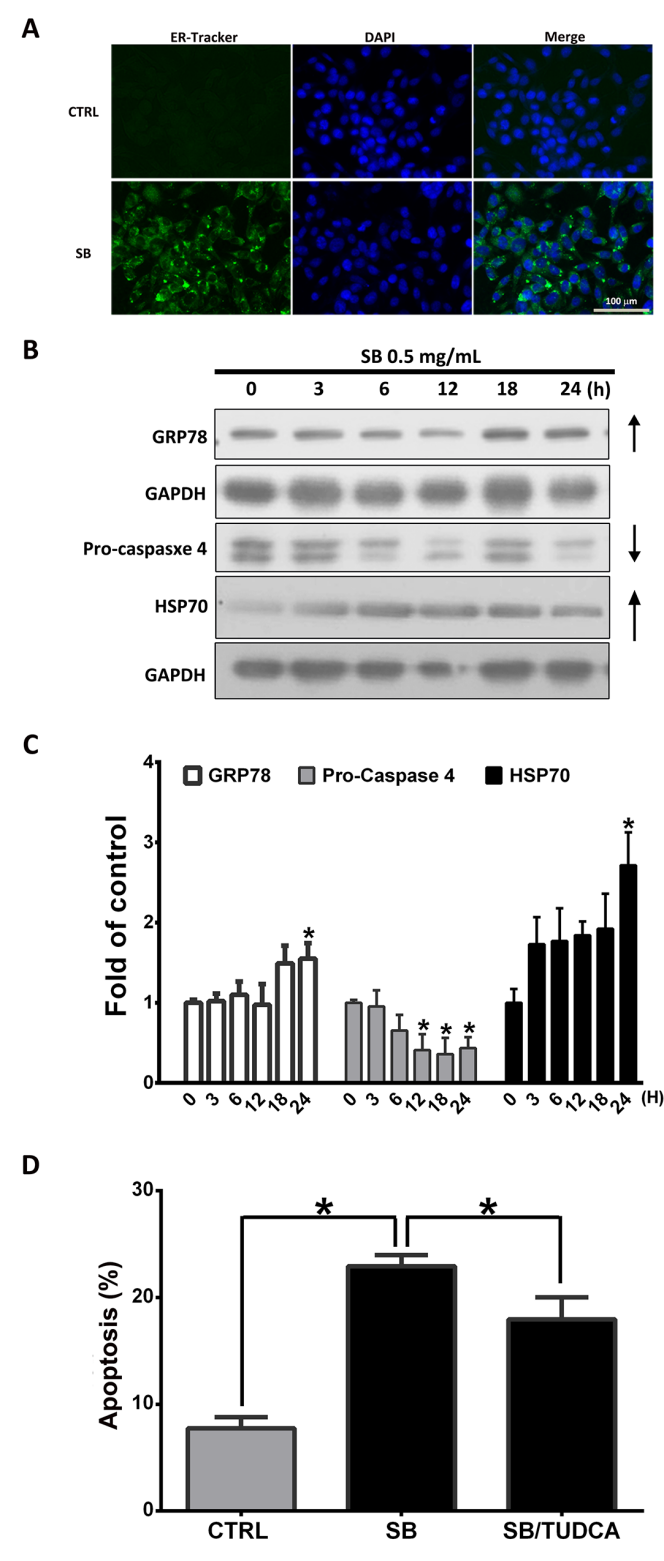
SB induces CL1-5 cell death through the pro-apoptotic ER Stress signaling pathway **(A)** Representative immunofluorescence images of ER-positive (green) CL1-5 cells at 24 h after exposure to 0.5 mg/ml SB. Green fluorescence intensity of the ER Tracker was increased in SB-treated cells compared with control cells. Cells were counterstained with DAPI (blue) to show all cell nuclei. Scale bar=100 μm. **(B)** Western blot analysis of pro-apoptotic and ER stress-related proteins after 0.5 mg/ml SB treatment of CL1-5 cells for different times. **(C)** Quantification of the western blot analysis. ^*^P<0.05 vs. the untreated group (CTRL). **(D)** CL1-5 cells were pre-incubated with or without TUDCA (10 μM) for 2 h and then incubated with SB for an additional 24 h. Cells were then stained with Annexin V–FITC and PI followed by flow cytometric analysis for apoptotic cell detection. Data are represented as the mean±SEM. N=4-7. ^*^P<0.05.

It has been reported that prolonged ER stress results in apoptosis [30, 31]. To demonstrate the role of ER stress in SB-induced apoptosis, an ER tracker stain was monitored in SB-treated CL1-5 cells. Representative microscopic images showed the increase in green fluorescence intensity and heterogenerous acculumation of ER-tracker in SB-treated CL1-5 cells relative to control cells (Figure 4A). *GRP78 and* HSP70 are ER-stress indicators when cells react with various stresses. Caspase 4 is a key player in the ER stress-mediated pathway of apoptosis. Western blot analysis showed that SB treatment for 0-24 h increased GRP78 and HSP70 expression, as well as caspase 4 activation, as evidenced by the reduction of procaspase 4 in CL1-5 cells in a time-dependent manner (Figure 4B-C). SB-induced apoptosis was significantly rescued after pretreatment with tauroursodeoxycholic acid (TUDCA; an ER stress inhibitor) compared with the SB treatment alone group (Figure 4D). Therefore, ER stress induced by SB may also play an important role in SB-induced CL1-5 cell apoptosis.

### SB induces mitochondrial-related and Fas-mediated apoptosis

**Figure 5:**
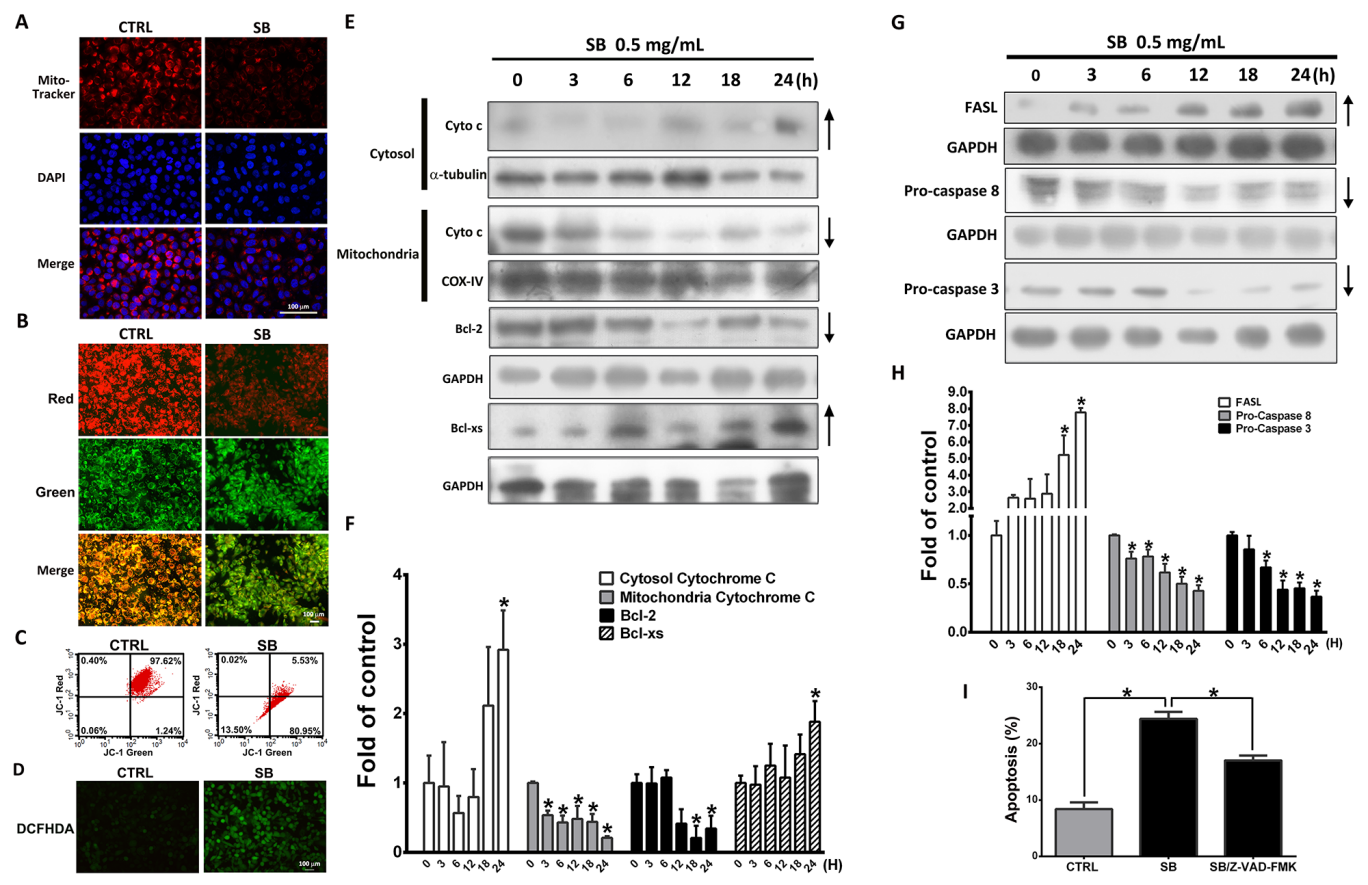
SB induces mitochondrial- and Fas-mediated apoptosis **(A)** Representative immunofluorescence images of mitochondria-positive (red) CL1-5 cells at 24 h after exposure to 0.5 mg/ml SB. Cells were counterstained with DAPI (blue) to show all cell nuclei. **(B)** Representative immunofluorescence images of JC-1 staining of CL1-5 cells at 24 h after exposure to 0.5 mg/ml SB. **(C-D)** Cells were treated with 0.5 mg/ml SB for 24 h and stained with H_2_DCFDA. The ROS intensity was detected and quantified by flow cytometry and immunofluorescence staining. **(E-F)** Western blot analysis and quantification of pro-apoptotic mitochondrial-related proteins after 0.5 mg/ml SB treatment of CL1-5 cells for different times. ^*^P<0.05 vs. the untreated group (CTRL). **(G-H)** Western blot analysis and quantification of pro-apoptotic FAS-related proteins after 0.5 mg/ml SB treatment of CL1-5 cells for different times. ^*^P<0.05 vs. the untreated group (CTRL). **(I)** Cells were pre-treated with 20 μM z-VAD-fmk for 1 h and then incubated with 0.5 mg/ml SB for 24 h. Cells were stained with Annexin V–FITC and PI followed by flow cytometric analysis for apoptotic cell detection. Data are represented as the mean±SEM. N=4-8. ^*^P<0.05. Scale bar=100 μm.

Apoptosis is mediated by intrinsic (or mitochondrial) and extrinsic (or death receptor) pathways [32]. To further examine whether SB-induced CL1-5 apoptosis is mediated by the mitochondrial pathway, CL1-5 cells were treated with 0.5 mg/ml SB for various times, and changes in the mitochondrial membrane potential were measured by Mito-Tracker and JC-1 staining. As shown in Figure 5A-B, SB treatment for 24 h decreased red fluorescence in the Mito-Tracker staining group and decreased the ratio of red fluorescence and green fluorescence by decreasing the intensity of red fluorescence in the JC-1 staining group compared with control cells. Similar data were observed in the JC-1 flow cytometry analysis (Figure 5C). These data indicated that mitochondrial function is critically impaired in SB-induced apoptosis in CL1-5 cells. Intracellular reactive oxygen species (ROS) generation is correlated with changes in mitochondrial membrane potential and is implicated in the induction of mitochondria-mediated apoptosis [33]. As shown in Figure 5D, ROS generation was detected following SB treatment. The key event of the mitochondrial apoptotic pathway is the loss of mitochondrial membrane potential and the release of cytochrome c from the mitochondria into the cytosol. Cytochrome c and the *Bcl*-*2 family* of *proteins* are important regulators of *mitochondria*-mediated apoptosis and caspase activation [34-36]. To determine the mechanisms of SB-induced mitochondria-related apoptosis, western blot analysis was performed. SB-treated CL1-5 cells had increased cytochrome c in the cytosolic fraction with decreased cytochrome c in the mitochondrial fraction (Figure 5E-F), and there were reduced levels of anti-apoptotic proteins (Bcl-2) and increased levels of pro-apoptotic proteins (Bcl-xs). Previous studies have shown that the FAS-FASL signaling pathway initiates the extrinsic apoptosis pathway through the activation of caspase 8- and caspase 3-mediated apoptotic cascades [37, 38]. As shown in Figure 5G-H, SB caused a significant time-dependent increase in FASL and a decrease in caspase 8 and caspase 3 expression. CL1-5 cells were pre-treated with z-VAD-fmk (a pan-caspase inhibitor) followed by SB treatment for an additional 24 h to investigate whether SB-induced apoptosis is dependent on mitochondria and death receptors. The Annexin V/PI staining showed that the number of apoptotic cells was significantly inhibited when both pathways were suppressed by z-VAD-fmk (Figure 5I). Taken together, these results strongly suggested that SB also induces CLI-5 cell apoptosis through mitochondria- and death receptor-mediated death receptor pathways.

### SB induces pro-apoptotic proteins by downregulating SIRT1 expression

**Figure 6:**
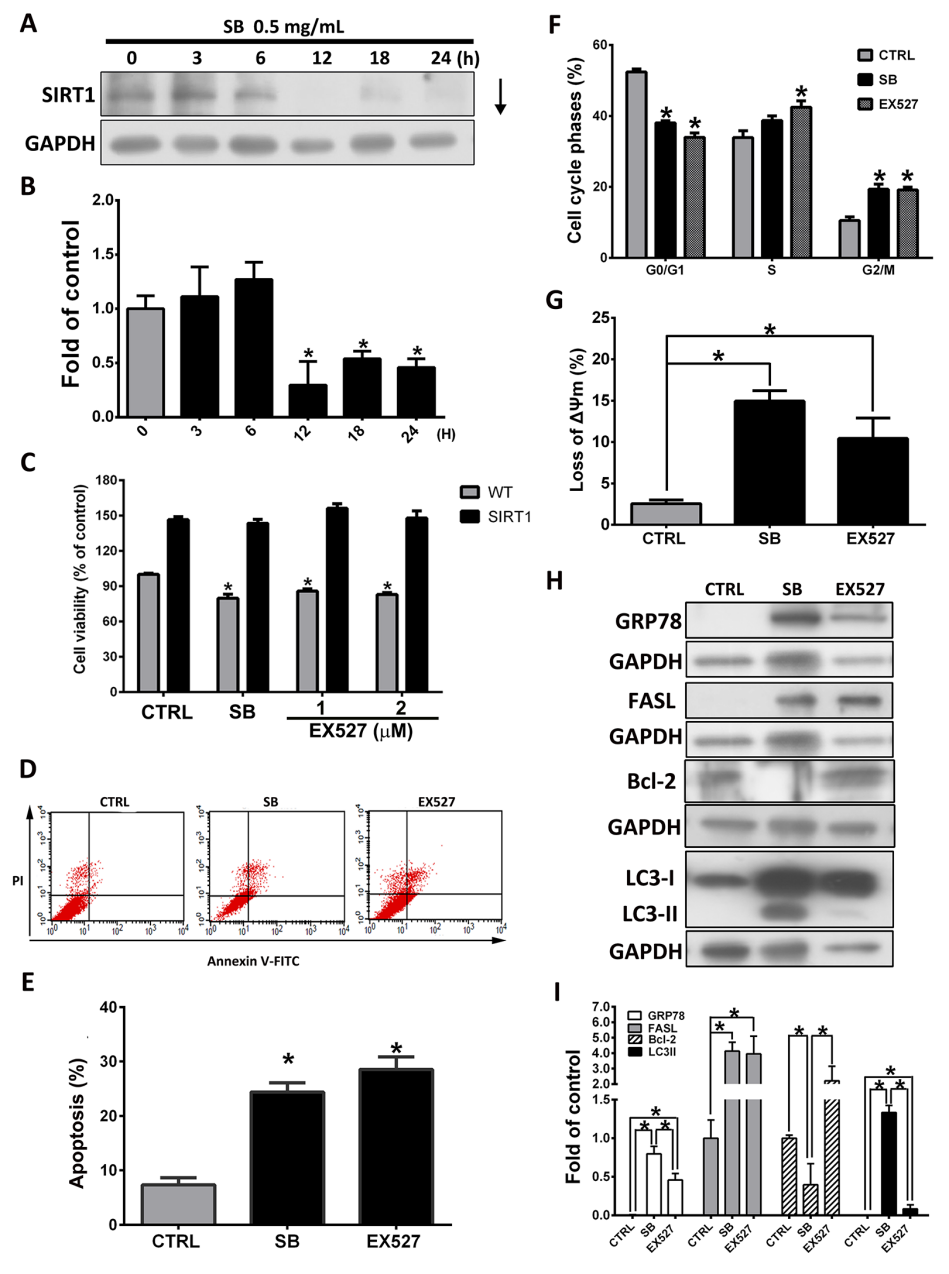
SB induces pro-apoptotic proteins by downregulating SIRT1 expression **(A)** Western blot analysis of SIRT1 after 0.5 mg/ml SB treatment of CL1-5 cells for different times. **(B)** Quantification of the western blot analysis. Cells were treated with SB or EX527 (1 or 2 μM) for 24 h. ^*^P<0.05 vs. the untreated group (CTRL). **(C)** CL1-5-empty- (WT) and CL1-5-SIRT1-overexpressing cells (SIRT1) were treated with SB or EX527 (1 or 2 μM) for 24 h and then cell viability was detected by MTT assay. ^*^P<0.05 vs. the untreated group (CTRL). **(D)** CL1-5 cells were treated with SB or EX527 (1 μM) for 24 h. Flow cytometry was used to determine the apoptotic cells. **(E)** Histogram shows the ratio of apoptotic cells induced by SB or EX527 treatment. ^*^P<0.05 vs. the untreated group (CTRL). **(F)** Flow cytometry was used to determine the cell cycle distribution. ^*^P<0.05 vs. the untreated group (CTRL). **(G)** Cells were treated with 0.5 mg/ml SB or 1 μM EX527 for 24 h and then stained with JC-1. The mean JC-1 fluorescence intensity was detected and quantified by flow cytometry. ^*^P<0.05. **(H-I)** Western blot analysis of GRP78, FASL and LC3II after 0.5 mg/ml SB or 1 μM EX527 treatment of CL1-5 cells for 24 h. Quantification of the western blot analysis. Data are represented as the mean±SEM. N=4-6. ^*^P<0.05.

Previous studies have shown that the expression of SIRT1 is associated with poor prognosis in lung cancer [39-41]. Inhibition of SIRT1 induces cell growth arrest, induces apoptosis and enhances chemosensitivity or radiation sensitization in lung cancer cells [11, 12, 42]. To further determine whether SB-mediated apoptosis occurs mainly through the suppression of the SIRT1 signaling pathway, CL1-5 cells were treated with 0.5 mg/ml SB for 0–24 h, and the expression of SIRT1 was assessed by western blot analysis. As shown in Figure 6A-B, SB treatment reduced SIRT1 expression in a time-dependent manner. To further determine whether SB-mediated apoptosis occurs mainly through the SIRT1 signaling pathway, CL1-5 cells were treated with the SIRT1-specific inhibitor, EX527, or SB for 24 h. As shown in Figure 6C, SB and EX527 significantly induced CL1-5 cell death, but the effect was not observed in SIRT1-overexpressing CL1-5 cells. Annexin V/PI staining (Figure 6D-E), cell cycle analysis (Figure 6F) and JC-1 staining (Figure 6G) also showed the presence of apoptotic cells, G2/M arrest and mitochondrial membrane potential (MMP) collapse in SB- or EX527-treated CL1-5 cells. As shown in Figure 6H-I, western blot analysis demonstrated that EX527 treatment significantly increased GRP78 and FASL expression but not LC3II expression. Therefore, we concluded that SB-induced CL1-5 cell apoptosis is regulated by cell cycle arrest, ER stress and the extrinsic-mediated pathway, and these effects were mainly mediated through the SIRT1 signaling pathway.

### SB induces CL1-5 cell death through activation of the P38 pathway

**Figure 7:**
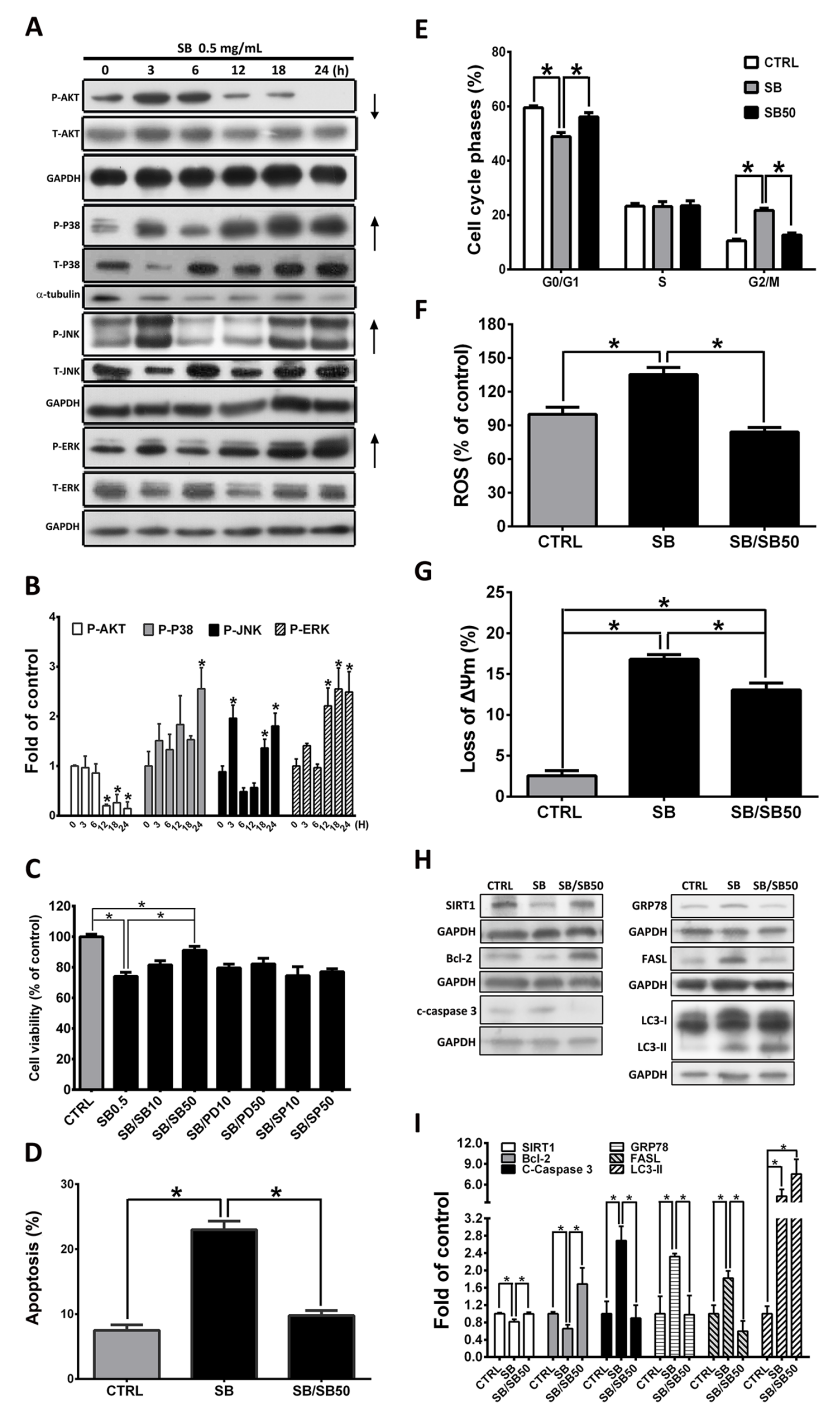
SB inhibits phosphorylation of MAPK in CL1-5 cells **(A)** CL1-5 cells were pretreated with 0.5 mg/ml SB for various times as indicated. The phosphorylated AKT, P38, JNK, and ERK levels were determined by Western blot analysis. GAPDH or α–tubulin was used as the loading control. **(B)** Quantification of the western blot analysis. ^*^P<0.05 vs. the untreated group (CTRL). **(C)** Cells were pre-treated with 10 or 50 μM SB203580 (SB10 or SB50), PD98059 (PD10 or PD50), or SP600125 (SP10 or SP50) for 1 h and then incubated with 0.5 mg/ml SB for 24 h. MTT assay was used to measure the cell viability of CL1-5 cells. **(D)** Flow cytometry was used to determine the apoptotic cells. Histogram shows the ratio of apoptotic cells induced by SB, and the effect was inhibited by SB50 pretreatment. **(E)** Flow cytometry was used to determine the cell cycle distribution. Cells were treated with 0.5 mg/ml SB or SB50 for 24 h and stained with H_2_DCFDA **(F)** and JC-1 **(G)**. The mean H_2_DCFDA and JC-1 fluorescence intensity was detected and quantified by flow cytometry. **(H-I)** Cells were pretreated with or without SB50 for 1 h and then 0.5 mg/ml SB for 24 h. The expression of SIRT1, Bcl-2, cleaved caspase 3, GRP78, FASL and LC3II was determined and quantified by western blot analysis. Data are represented as the mean±SEM. N=4-7. ^*^P<0.05.

Previous studies have shown that AKT and mitogen-activated protein kinase (MAPK) play important roles in cell proliferation and apoptosis [43-45]. The effects of the expression and phosphorylation of AKT and MAPKs on SB-treated CL1-5 cells were examined. As shown in Figure 7A-B, 0.5 mg/ml SB treatment significantly increased the phosphorylation of P38, JNK and ERK but decreased the phosphorylation of AKT in a time-dependent manner. To further determine whether SB-mediated apoptosis occurs mainly through the activation of the MAPK signaling pathway, CL1-5 cells were pretreated with a MAPK-specific inhibitor for 1 h followed by treatment with SB for 24 h. As shown in Figure 7C-D, only pretreatment with the 50 μM P38 inhibitor, SB203580 (SB50), significantly restored cell death and apoptosis in response to SB treatment, and the ERK- and JNK-specific inhibitors did not prevent SB-induced apoptosis. SB-induced G2/M phase arrest, ROS, and MMP collapse were significantly restored by 50 μM SB203580 (SB50) pretreatment (Figure7E-G). As shown in Figure 7H-I, western blot analysis revealed that SB203580 pretreatment significantly reversed the changes in expression of SIRT1, Bcl-2, cleaved caspase 3, GRP78 and FASL in SB-treated CL1-5 cells, but SB203580 pretreatment did not affect LC3II expression. Taken together, these results strongly suggested that SB-induced CL1-5 cell apoptosis is regulated by cell cycle arrest, ER stress, the intrinsic-mediated pathway and the extrinsic-mediated pathway, and these effects were exerted mainly through the P38 signaling pathway.

### SB induces autophagy in CL1-5 cells

**Figure 8:**
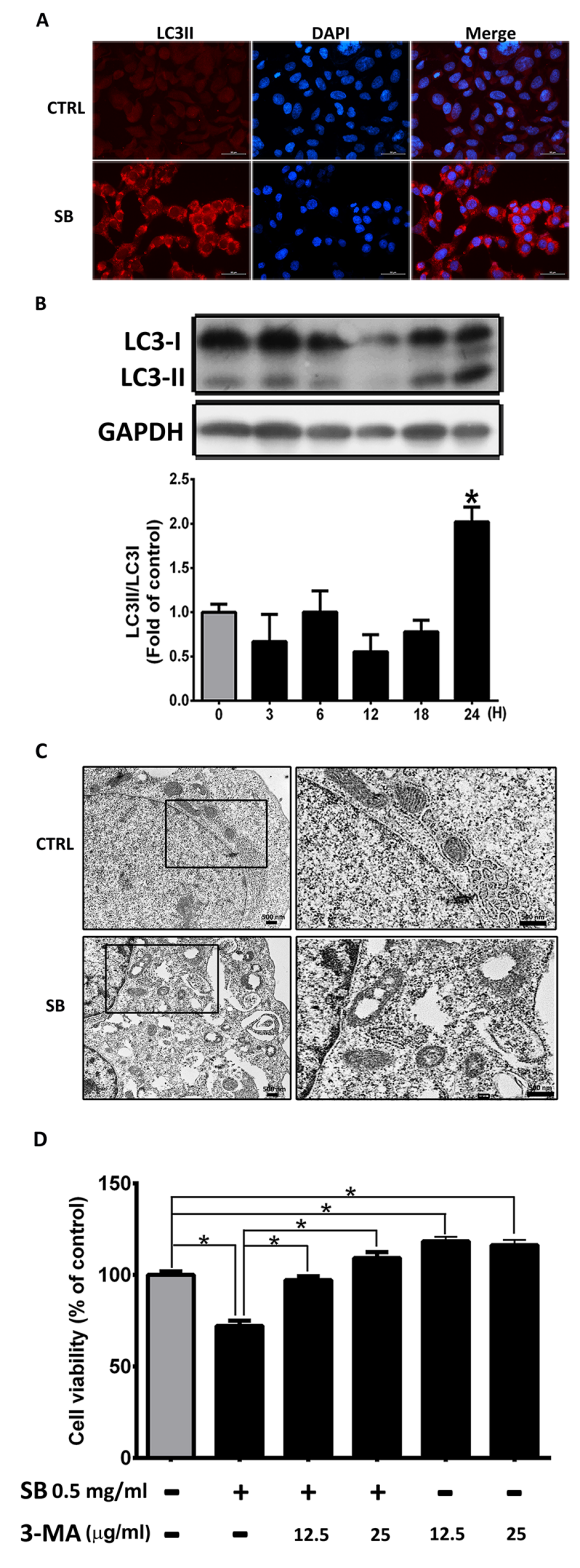
SB induces autophagy in CL1-5 cells **(A)** Representative immunofluorescence images of LC3II-positive (red) CL1-5 cells at 24 h after exposure to 0.5 mg/ml SB. The cells were counterstained with DAPI (blue) to show all cell nuclei. Scale bar=50 μm. **(B)** Western blot analysis of LC3II after 0.5 mg/ml SB treatment of CL1-5 cells for different times. ^*^P<0.05 vs. the untreated group (CTRL). **(C)** TEM images of CL1-5 cells treated with vehicle (CTRL) or SB after 24 h. Higher magnification images (right panel) illustrate autophagic vacuoles (arrow) in SB treated-CL1-5 cells. Scale bar=500 nm. **(D)** MTT assay was used to measure the viability of CL1-5 cells at 24 h after exposure to SB and/or 3-MA. Data are represented as the mean±SEM. N=4-6. ^*^P<0.05.

Previous studies have shown that apoptosis and autophagy are important molecular processes that maintain organismal and cellular homeostasis, respectively. To demonstrate the role of autophagy in SB-induced death, the levels of LC3II were measured in SB-treated CL1-5 cells by LC3II staining, western blot analysis, and TEM. As shown in Figure 8A-B, SB caused a significant increase in LC3II. Large and numerous autophagic vacuoles, dilated ER, and injured mitochondria were observed in SB-treated CL1-5 cells (Figure 8C). With 3-MA (an autophagy inhibitor) co-treatment, SB-induced CL1-5 cell death was inhibited (Figure 8D). Taken together, these results strongly suggested that SB also induces CL1-5 cell death through autophagy.

### SB suppresses tumor growth in mouse xenograft models

**Figure 9:**
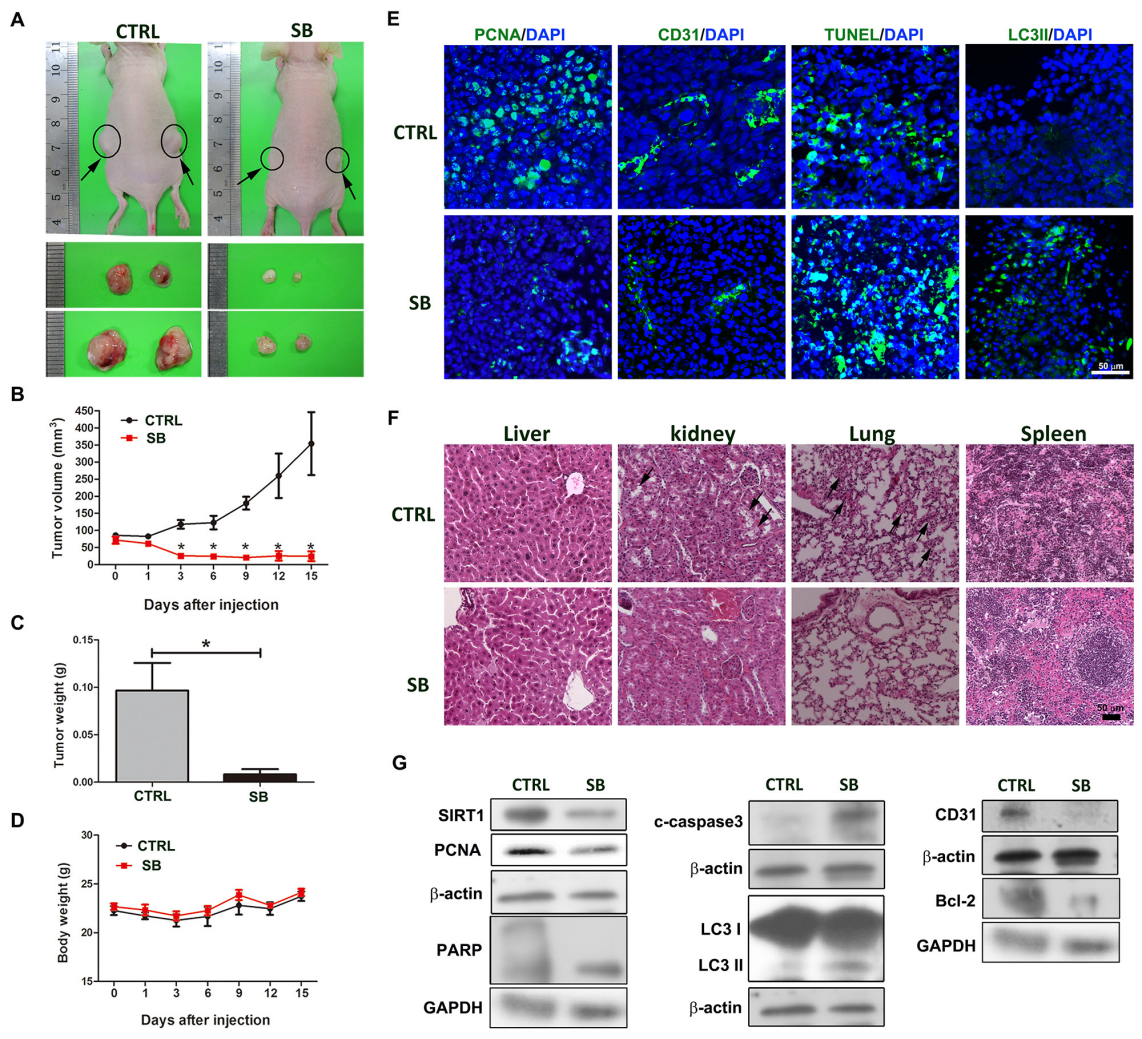
SB suppresses tumor growth in mouse xenograft models **(A-C)** CL1-5 cells were injected subcutaneously into nude mice. The mice were treated with i.p. injections of either control or SB (60 mg/kg) six times per week. Tumor sizes were recorded every 2 days. After treatment for 15 days, mice were sacrificed, and tumors were collected, photographed and weighed. **(D)** The body weight was also recorded. **(E)** Immunohistochemical staining with antibodies against PCNA, CD31, TUNEL and LC3II in tumor sections. Scale bar=50 μm. **(F)** Representative cross-sections of the liver, kidney, lung and spleen were stained with hematoxylin and eosin. Arrows indicated cell loss or inflammatory cell infiltration in untreated kidney or lung tissue. Scale bar=50 μm. **(G)** The tumors from control and SB-treated mice were harvested for western blot analysis. Data are represented as the mean±SEM. N=5-6. ^*^P<0.05 vs. the untreated group (CTRL).

To further demonstrate the anti-tumor effect of SB on CL1-5 cells, a mouse xenograft model was used. CL1-5 cells (1.5x10^6^) were subcutaneously implanted in the back flank of nude mice, and SB was administered at 60 mg/kg by intraperitoneal injection. Figure 9A-C shows that SB treatment markedly inhibited tumor growth as evidenced by reduced tumor mass. As compared with the vehicle group, no negative effect was observed in SB-treated mice according to the body weight measurement (Figure 9D). These results showed that SB is an effective anti-tumor agent. PCNA, CD31, TUNEL assay, and LC3II staining showed fewer proliferating tumor cells and CD31-positive vessel cells in addition to more dead cells in SB-treated tumor tissues (Figure 9E). Major organs of the mice were histologically examined after intraperitoneal injection of SB treatment. After H&E staining, the tissue slices (liver, kidney, lung, and spleen) were observed 15 days post-treatment, and SB-treated tissue did not show severe damage, indicating low toxicity in these organs after SB treatment. By contrast, increased inflammatory cell infiltration, cell loss and tissue damage were observed in the untreated lungs and spleens obtained from CL1-5 tumor cell-bearing mice (Figure 9F). Western blot analysis showed that the levels of SIRT1, PCNA, CD31, and Bcl-2 were decreased by SB treatment, and the levels of cleaved PARP, cleaved caspase 3 and LC3II were increased by SB treatment (Figure 9G). These data showed that the SB-induced anti-tumor effects occurred through apoptosis, autophagy, anti-angiogenesis and anti-proliferation. These effects were similar to those of SB-treated CL1-5 cells *in vitro*.

### SB increases the therapeutic effects of etoposide or cisplatin treatment in CL1-0, CL1-5 and A549 cells

**Figure 10:**
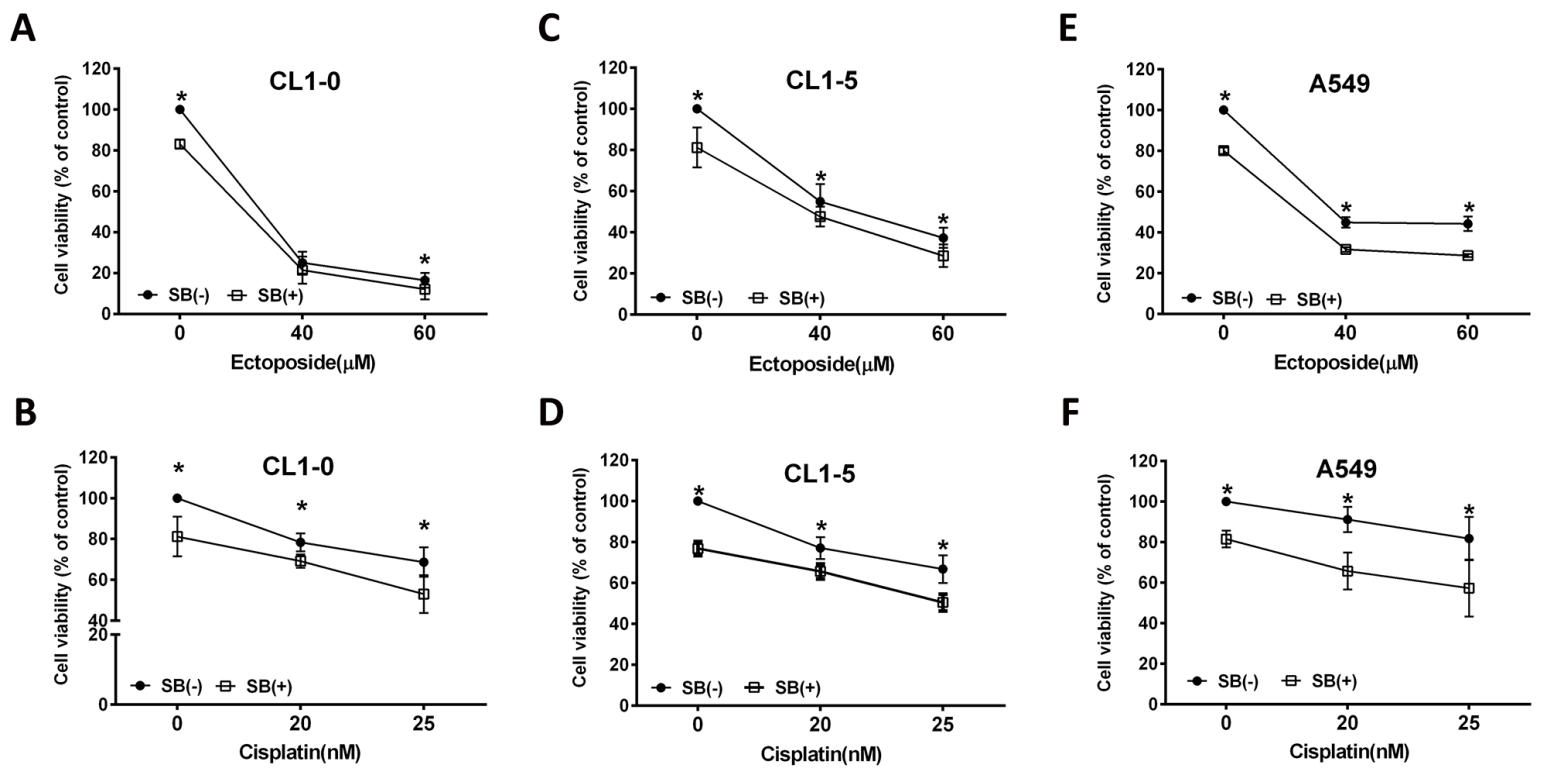
SB increases the chemosensitivity of CL1-0, CL1-5 and A549 cells to etoposide **(A, C, E)** or cisplatin **(B, D, F)** treatment. Data are represented as the mean±SEM. N=4-12. *P<0.05 vs. the SB-treated group.

In addition to surgery, chemotherapy is the most common strategy used to treat cancer. However, high toxicity and drug resistance hamper and restrict the therapeutic effects of chemotherapeutic drugs. In the present study, combinations of SB and several chemotherapeutic drugs, including etoposide and cisplatin, suppressed cell growth more than each drug treatment alone (Figure 10). These results suggested that SB exerts additive effects when combined with etoposide or cisplatin on lung cancer cells.

## DISCUSSION

SB is a well-known herb and drug in traditional Chinese Medicine in which it is used as an anti-inflammatory and an antitumor agent. In the present study, we showed that SB exerts anti-tumor effects both *in vitro* and *in vivo*. The apoptosis of SB-treated CL1-5 cells is controlled by the cell cycle and ER stress-, intrinsic mitochondrial-, and extrinsic FAS/FASL-mediated pathways. These anticancer effects of SB are mainly mediated by the P38/SIRT1 pathway, leading to G2/M phase arrest and apoptosis in CL1-5 cells. The SB-induced CL1-5 cell cytotoxicity is partly attributed to autophagy. In addition, SB increases the therapeutic effects of etoposide or cisplatin treatment on lung cancer cells. These data indicated that SB may be a potential and effective anti-lung cancer drug.

Cell proliferation is highly dysregulated in cancer cells, and cell proliferation is controlled by the cell cycle. Cell cycle progression delay or arrest is related to apoptosis. The mechanism of anti-cancer drugs is to delay the cell cycle progression of tumor cells by regulating checkpoints, inducing cell arrest and then initiating cell apoptosis. It has been reported that SB induces cancer cell apoptosis through interfering with cell cycle progression by G1/S [46, 47] or G2/M arrest [48]. In the present study, SB caused CL1-5 cell G2/M arrest by reducing the expression levels of Cdc25C, cyclin A, cyclin B1, and Cdc2. Taken together, these data showed that SB causes cell arrest and apoptosis in cancer cells. These results suggested that the anti-cancer mechanism of SB is mainly mediated by inducing G2/M arrest in CL1-5 cells.

The *ER* plays an important role in the regulation of cellular responses to stimuli and calcium homeostasis [49]. Accumulation of misfolded proteins in the ER causes ER stress. GRP78, an ER chaperone protein, and HSP70 are upregulated by ER stress [50]. Caspase 4 is activated by ER stress and is involved in ER stress-induced apoptosis [51, 52]. In the present study, SB treatment increased the expression of GRP78 and HSP70, as well as caspase 4 activation, as evidenced by the reduction of procaspase 4 in CL1-5 cells in a time-dependent manner (Figure 4B-C). TUDCA pretreatment partially reduced SB-induced apoptosis (Figure 4D). Therefore, we concluded that ER stress may also play a crucial role in SB-induced CL1-5 cell apoptosis.

Apoptosis plays an important role in development and organismal homeostasis maintenance. Defective or dysregulated apoptosis results in cancer, degenerative diseases and autoimmunity [53]. Induction of cell *apoptosis* and regulation of apoptosis-inducing pathways have been an effective *strategy* for *cancer* therapy. Two main apoptotic pathways include the intrinsic mitochondrial- and extrinsic death receptor-mediated pathways. The intrinsic mitochondrial pathway involves mitochondrial permeability transition pore (MPTP) disruption, resulting in immediate MMP collapse, cytochrome c release, caspase 3 activation, change in pro-apoptotic and anti-apoptotic protein ratios and initiation of the mitochondrial apoptosis cascade. The extrinsic pathway involves the interaction of the death receptor and its ligand, resulting in caspase 8 activation, which then triggers the apoptosis cascade. In our study, MMP collapse was observed after SB treatment (Figure 5B-C). In addition, western blot analysis also showed that SB induced cytochrome c release, caspase 3 activation, caspase 8 activation, pro-apoptotic protein (Bcl-xs) upregulation, FASL upregulation and anti-apoptotic protein (Bcl-2) downregulation (Figure 5E-H). Furthermore, we pretreated cells with z-VAD-fmk (a pan-caspase inhibitor) to suppress caspase cascade activation in both the intrinsic mitochondrial- and the extrinsic death receptor-mediated pathways. The high apoptotic percentage induced by SB was suppressed by z-VAD-fmk pretreatment (Figure 5I). These data suggested that SB-induced CL1-5 cell apoptosis occurs through both the intrinsic mitochondrial- and the extrinsic death receptor-mediated pathways.

Autophagy, characterized by double-membrane autophagosome accumulation in the cytosol, is an intracellular catabolic mechanism to maintain cell homeostasis. The major function of autophagy is to remove lysosome-mediated degradation of damaged proteins and to protect organisms against various stress and stimuli. However, the role of autophagy in cancer cell death is still controversial. In the present study, immunostaining, western blot analysis and TEM showed that LC3II expression and autophagic vacuoles were induced by SB treatment in CL1-5 cells. The MTT assay showed that SB-induced CL1-5 cell death was markedly reversed by 3-MA treatment, and cell viability was significantly increased by 3-MA treatment alone compared with the control group (Figure 8D). Our data revealed that autophagy plays a role in SB-induced CL1-5 cell death and serves as a cell destructive pathway.

SIRT1, a NAD+-dependent protein deacetylase, regulates cell growth, apoptosis, aging and cellular metabolism through deacetylation and the regulation of multiple proteins, including p53, PGC-1α, FOXO, NF-κB, histones and DNA damage response proteins [6-8, 54, 55]. A previous study showed that SIRT1 expression is associated with the poor prognosis of lung cancer and that SIRT1 induces cancer cell apoptosis via the modulation of p53 and FOXO [6]. However, the regulatory role of SIRT1 in SB-treated CL1-5 cells was not clear. The present data showed that inhibition of SIRT1 by EX527 treatment enhanced CL1-5 cell apoptosis, and this effect was similar to that resulting from SB treatment alone (Figure 6C-E). In addition, cytotoxicity was reversed by the overexpression of SIRT1 (Figure 6C). In addition, the G2/M arrest, MMP collapse, GRP78 induction and FASL activation resulting from SB treatment were also observed in the EX527 treatment alone group. The present study demonstrated that the SB-induced CL1-5 cell apoptosis was mediated by SIRT1.

Previous studies have shown that MAPK and AKT are involved in the signal transduction pathways that lead to the regulation of the cell cycle, cellular stress response and apoptosis. Several studies have shown that SB induces cancer cell apoptosis by regulating the AKT, *Signal transducer and activator of transcription 3* (*STAT3*), ERK, *JNK* and P38 pathways [17, 56, 57]. In the present study, our data demonstrated that the P38 MAPK inhibitor, SB203580, reversed the apoptosis, G2/M arrest, ROS, and MMP collapse in SB-treated CL1-5 cells (Figure 7D-G). However, SB-induced autophagy was not influenced by P38 MAPK inhibition (Figure 7H-I). Therefore, we concluded that the activation of P38 MAPK may be involved in SB-induced cell cycle arrest, apoptosis, and ROS in CL1-5 cells.

*Scutellaria barbata* D. Don (SB), a herb with anti-inflammation and anti-cancer effects, is used to treat various types of cancer, including colon, breast, gastric, liver and lung cancer. However, the precise regulatory mechanism of SB treatment on lung cancer is still unclear. Previous studies have revealed that SB inhibits 95-D cell invasion and migration via inhibition of the C-met pathway [26]. SB inhibits Calu-3 cell growth via suppression of the HER2 pathway and angiogenesis [28]. SB induces A549 cell apoptosis through the intrinsic mitochondrial pathway [58]. In our *in vitro* study, SB-induced CL1-5 cell death occurred through multiple pathways, including the intrinsic mitochondrial-, extrinsic death receptor-, and ER stress-mediated apoptosis pathways, as well as through cell cycle arrest and autophagy. Our *in vivo* study demonstrated that SB decreased growth and angiogenesis, as well as enhanced apoptosis and autophagy in tumors.

**Figure 11:**
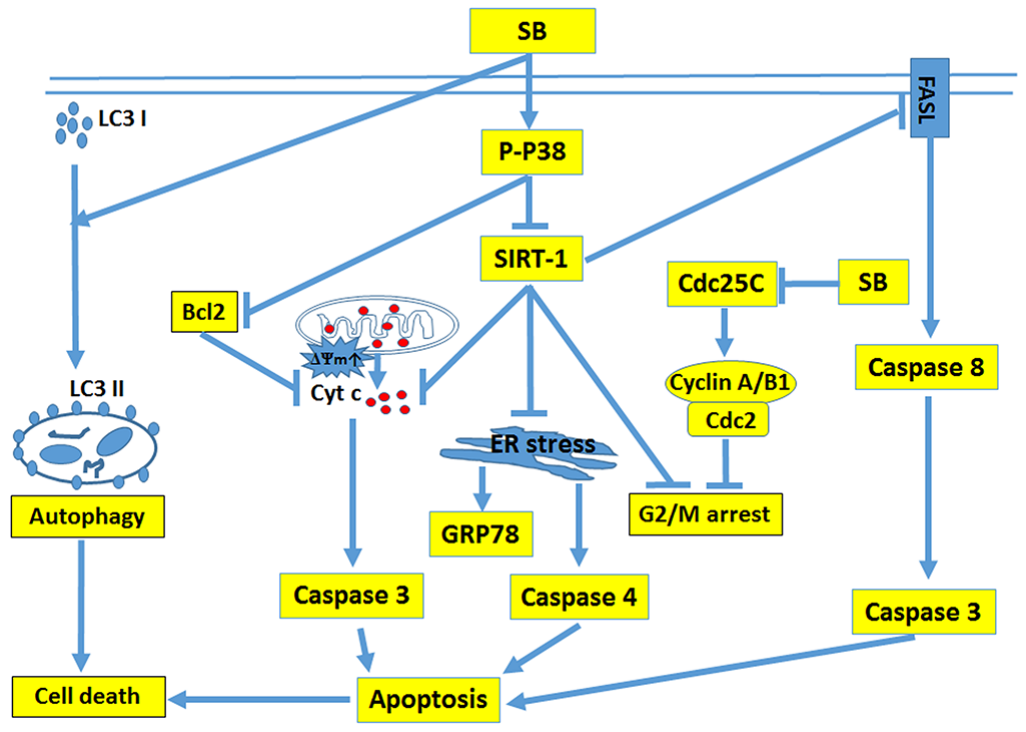
Graphical scheme of the anti-cancer mechanism of SB in CL1-5 cells

The results of the present study clearly showed that SB exerted anti-tumor effects by increasing CL1-5 cell death through multiple pathways (Figure 11). SB induced CL1-5 cell death through the modulation of P38/SIRT1, which induced cell cycle arrest and the apoptosis pathway *in vitro*. SB also suppressed tumor growth and promoted apoptosis and autophagy in tumors *in vivo*. In addition, SB exerted additive effects when combined with etoposide or cisplatin treatment of lung cancer cells. These results indicated that SB is a good candidate for use as an anti-lung cancer agent.

## MATERIALS AND METHODS

### Reagents

Polyclonal rabbit IgG antibodies against human GAPDH, α-tubulin, LC3II, Bcl-2, Bcl-xs, Cdc2, Cdc25C, phospho-/total-P38, phospho-/total-ERK, phospho-/total-JNK and phospho-/total-AKT, as well as horseradish peroxidase (HRP)-conjugated goat anti-mouse IgG, and anti-rabbit IgG antibodies were purchased from GeneTex (Irvine, CA, USA). Monoclonal rabbit antibodies against human proliferating cell nuclear antigen (PCNA), Cyclin B1 and Cyclin A were purchased from Santa Cruz Biotechnology (Santa Cruz, CA, USA). Polyclonal rabbit IgG against human PARP, Caspase 3, Caspase 4, Caspase 7, Caspase 8, FASL, Glucose-regulated protein 78 (*GRP78)*, Heat shock protein 70 (HSP70) and Sirtuin 1 (SIRT1) were purchased from Cell Signaling Technology (Beverly, MA, USA). CD31, COX IV, and Cytochrome C antibodies were purchased from Abcam (Cambridge, UK). PD98058, SP600125, and SB203580 were purchased from Biomol (Plymouth Meeting, PA, USA). 3- (4,5-cimethylthiazol-2-yl)-2,5-diphenyl tetrazolium bromide (MTT), JC-1, H_2_DCFHDA and *Propidium iodide* (PI) were purchased from Sigma-Aldrich (St. Louis, MO, USA). Mitotracker and ER tracker were purchased from Molecular Probes (Invitrogen, Carlsbad, CA, USA). TUNEL assay Kit and Annexin V/PI Staining Kit were purchased from Roche (*Applied Science*, Germany). TRITC- and FITC-conjugated goat anti-mouse IgG antibodies were purchased from Jackson ImmunoResearch (West Grove, PA, USA).

### Preparation of *Scutellaria barbata* D. Don (SB) extract

SB was purchased from the market in Taichung and identified by Dr. Kao Chun-Pin. Briefly, SB was extracted with methanol and then filtered. The filtrate was concentrated to dryness under reduced pressure to afford a crude extract, which was analyzed by HPLC (Figure 1A). The extracted fraction was stored at -20°C until further use.

### Cell culture

Human lung cancer cell lines, including A549, CL1-0, and CL1-5, were obtained from American Type Culture Collection (ATCC). Cells were grown in RPMI-1640 medium (GIBCO) supplemented with 10% fetal bovine serum, 100 units/ ml penicillin and 100 Ag/ml streptomycin at 37°C in an incubator containing 5% CO2.

### MTT assay

Cells were seeded at a concentration of 1.5x10^4^ cells/ml in a 96-well plate. After treatment with 0.5 mg/ml SB for 24 h, cell viability was determined by the MTT assay.

### Annexin V/PI staining

Annexin V/PI Staining was performed as described by the manufacturer. Apoptotic cells were visualized by fluorescence microscopy and expressed as a percentage of apoptotic cells. Briefly, CL1-5 cells were cultured in a 6-well culture plate. After treatment with 0.5 mg/ml SB for 24 h, the cells were trypsinized and stained with staining solution at room temperature for 10-15 min in the dark. The cell apoptotic ratio was detected by a FACScan cytometer (BD Biosciences, San Jose, CA, USA) and was analyzed by ModFit software.

### Cell cycle analysis

CL1-5 cells were cultured in a 6-well culture plate. After treatment with 0.5 mg/ml SB for 24 h, the cells were trypsinized and stained with PI. The cell cycle progression was detected on a FACScan cytometer and was analyzed by ModFit software.

### TUNEL assay

DNA fragmentation of the cells *in situ* was detected by a TUNEL assay according to the manufacturer’s instructions. Briefly, after treatment with 0.5 mg/ml SB for 24 h, CL1-5 cells were fixed in 4% paraformaldehyde for 15 min and then incubated with 0.1% Triton X-100 in PBS for 15 min at room temperature. After washing in PBS, the cells were incubated with TUNEL reaction buffer for 60 min at 37°C, counterstained with DAPI and observed with fluorescence microscopy.

### Mitochondrial and endoplasmic reticulum (ER) activity detection

Mitochondrial and endoplasmic reticulum (ER) activities were detected by staining with Mito-Tracker Red and ER-Tracker Green, respectively, following the manufacturer’s instructions. CL1-5 cells (2 × 10^4^ cells/well) were seeded on glass coverslips and treated with 0.5 mg/ml SB for 24 h. After SB treatment, the cells were treated with 1 μM Mito-Tracker or 1 μM ER-Tracker for 60 min. The cells were fixed in 4% paraformaldehyde, counterstained with 1 μg/mL DAPI and observed with fluorescence microscopy.

### Mitochondria membrane potential assay

JC-1 was used to detect the mitochondria membrane potential. CL1-5 cells were treated with 0.5 mg/ml SB for 24 h, and the cells were then incubated with 10 μM JC-1 at 37 °C for 30 min. After incubation, the cells were washed with warm PBS buffer and observed with a fluorescence microscope.

### Measurement of ROS generation

H_2_DCFDA was used to detect ROS generation. CL1-5 cells were treated with 0.5 mg/ml SB for 24 h, and the cells were then incubated with 10 μM H_2_DCFDA at 37 °C for 30 min. After incubation, the cells were washed with warm PBS buffer and observed with a fluorescence microscope.

### Immunoblotting analysis

Western blot analysis was performed as described previously [59]. Briefly, CL1-5 cells were treated with 0.5 mg/ml SB for various treatment times and then lysed with lysis buffer (RIPA; Cell Signaling Technology, Beverly, MA, USA). Proteins (30 μg) were subjected to SDS–PAGE with 10-12% polyacrylamide gradient gels and transferred onto PVDF membranes (Millipore, Bedford, MA, USA). The membranes were blocked with 5% BSA at room temperature for 1 h. The blots were incubated with primary antibodies (all 1:1000 in 1.5% BSA) at 4°C overnight and then incubated with HRP-conjugated secondary antibodies (all 1:6000) at room temperature for 1 h. Immunoreactivity was detected with ECL (GE Healthcare Bioscience). Intensities of bands were quantified using gel-pro software. Rabbit antihuman GAPDH, α–tubulin and β–actin antibodies were used as internal controls (all 1:10000 in 1.5% BSA). The protein amounts were normalized by the intensities of the internal control bands.

### SIRT1 overexpression

Overexpression of SIRT1 gene expression was performed by transfection with a SIRT1 plasmid. CL1-5 cells (5x10^6^ cells) were added to 3 ml of plasmid (0.25 μg)–GenJet complex (GenJet, US). The cells were used for experiments 24 h after transfection.

### Transmission electron microscope

Cells were fixed in 2% glutaraldehyde and 2% paraformaldehyde for 24 h, and cells were then treated with 1% osmium tetroxide for 1 h. Cells were subsequently embedded in Epon and processed for TEM (HITACHI H-700).

### *In vivo* xenograft model

For *in vivo* tumor growth studies, 1.5 × 10^6^ CL1-5 cells in a volume of 100 μl were injected subcutaneously into the lower flanks of BALB/c nude mice (National Laboratory Animal Center). SB treatment (60 mg/kg) was initiated when the tumors were 100 mm^3^ in volume. Mice were divided into two groups and randomly treated with the compound being tested or vehicle (both injected i.p. six times per week). Tumor volume was calculated according to the following formula: volume = length x width^2^ x 0.52. All procedures were performed in accordance with the local institutional guidelines for animal care of the National Taiwan University and complied with the “Guide for the Care and Use of Laboratory Animals” NIH publication No. 86-23, revised 2011. After 15 days of treatment with SB or vehicle, the mice were sacrificed by an overdose of pentobarbital, and the tumors were collected and weighed. Half of the tumors were collected for analysis of protein expression. The remaining tumors, kidneys, lungs, spleens and livers were fixed in 4% paraformaldehyde, paraffin-embedded and cross-sectioned for morphometric analysis and immunohistochemistry.

### Statistics

All values are presented as the mean ± standard error mean (mean ± SEM). All study data were analyzed using an analysis of variance followed by Turkey’s test for pairwise comparison. Significance was defined as *P-*values*<*0.05 for all tests. Each value was tested in triplicate.
